# Fibrin Glue Implants Seeded with Dental Pulp and Periodontal Ligament Stem Cells for the Repair of Periodontal Bone Defects: A Preclinical Study

**DOI:** 10.3390/bioengineering8060075

**Published:** 2021-06-01

**Authors:** Natella I. Enukashvily, Julia A. Dombrovskaya, Anastasia V. Kotova, Natalia Semenova, Irina Karabak, Roman E. Banashkov, Dmitry Baram, Tatiana Paderina, Stanislav S. Bilyk, Wolf-Dieter Grimm, Anton N. Kovalenko, Dmitry Ivolgin, Egor M. Prikhodko, Alexey V. Silin

**Affiliations:** 1 Cell Technologies Lab, North-Western State Medical University, 191015 St. Petersburg, Russia; anastkotova@gmail.com (A.V.K.); prof_wolf.grimm@yahoo.de (W.-D.G.); Dmitrii.Ivolgin@szgmu.ru (D.I.); 2 Institute of Cytology of the Russian Academy of Sciences, 194064 St. Petersburg, Russia; 3 General Dentistry Department, North-Western State Medical University, 191015 St. Petersburg, Russia; paderina_98@list.ru (T.P.); a.silin@szgmu.ru (A.V.S.); 4 Russian Research Institute of Hematology and Transfusiology, FMBA of Russia, 191024 St. Petersburg, Russia; semenova@mlc-lab.ru (N.S.); bdv150595@yandex.ru (D.B.); 5 Children’s Scientific and Clinical Center for Infectious Diseases, 197022 St. Petersburg, Russia; irina-karabak@mail.ru; 6 X-ray Centers «Picasso», 191123 St. Petersburg, Russia; picasso.spb.doctor@gmail.com; 7 Vreden National Medical Research Center of Traumatology and Orthopedics, 195427 St. Petersburg, Russia; bss0413@gmail.com (S.S.B.); tonnchik@ya.ru (A.N.K.); 8 Periodontology, Faculty of Health, School of Dental Medicine, Witten/Herdecke University, 58455 Witten, Germany; 9 Cell Technologies Center Pokrovsky, LLC, 199106 St. Petersburg, Russia; ceo@pokrovcell.ru; 10 Therapeutic Department, North-Western State Medical University, 191015 St. Petersburg, Russia

**Keywords:** scaffold, dental pulp stem cells, 3D printed scaffold mold, bone defect, fibrin glue, cell technologies in regenerative dentistry, computed tomography

## Abstract

A technology to create a cell-seeded fibrin-based implant matching the size and shape of bone defect is required to create an anatomical implant. The aim of the study was to develop a technology of cell-seeded fibrin gel implant creation that has the same shape and size as the bone defect at the site of implantation. Using computed tomography (CT) images, molds representing bone defects were created by 3D printing. The form was filled with fibrin glue and human dental pulp stem cells (DPSC). The viability, set of surface markers and osteogenic differentiation of DPSC grown in fibrin gel along with the clot retraction time were evaluated. In mice, an alveolar bone defect was created. The defect was filled with fibrin gel seeded with mouse DPSC. After 28 days, the bone repair was analyzed with cone beam CT and by histological examination. The proliferation rate, set of surface antigens and osteogenic potential of cells grown inside the scaffold and in 2D conditions did not differ. In mice, both cell-free and mouse DPSC-seeded implants increased the bone tissue volume and vascularization. In mice with cell-seeded gel implants, the bone remodeling process was more prominent than in animals with a cell-free implant. The technology of 3D-printed forms for molding implants can be used to prepare implants using components that are not suitable for 3D printing.

## 1. Introduction

A modern multidisciplinary approach for bone tissue regeneration has been developed in recent years. Tissue engineering and 3D technologies have made it possible to create tools for designing three-dimensional structural and functional matrices–scaffolds that can be molded to match the shape and size of a bone defect [1]. The use of three-dimensional scaffolds allowed to achieve a successful result not only due to filling the area of the defect with some bone substitutes but also due to the stimulation of local regeneration processes that contribute to the complete restoration of function [2,3].

The selection of appropriate three-dimensional scaffolds from biocompatible materials is one of the most important goals in regenerative dentistry. These scaffolds are required to provide optimal conditions for cell growth and differentiation, vascularization and remodeling of regenerating bone tissue as well to be non-toxic for a patient [4]. Scaffolds, which match a defect shape and are made of biocompatible (and often tissue-specific) materials, can integrate into a patient’s tissues. They are a source of growth factors and an additional area of cell adhesion and provide a proper milieu for cell attachment, proliferation and functioning.

Dental pulp stem cells (DPSC) are cranial neural crest-derived stem cells present in dental pulp [5,6,7]. Multipotent and pluripotent dental pulp stem cells (DPSC) are capable of differentiation into cells of the tissues of the tooth and periodontium [5,7,8,9]. They can also interact with neighboring cells, promoting regeneration and downregulating inflammation [5,6]. Unlike other cells of the mesenchymal phenotype, DPSC, as well as other stem cells from ligamentum, apical papilla, dental follicle and gingiva, are of ectodermal origin [5]. Some features of these cells are of great importance for dentistry: the formation of pulp-like tissue, the ability for differentiation into odontoblast-like cells, the synthesis of dentin- and cementum-like extracellular matrix during general osteogenic differentiation as well as during differentiation into odontoblasts [5]. All of these capabilities make neural-crest derived cells a promising tool for regenerative dentistry [5,6,10].

To function effectively, DPSC should be combined with a scaffold, which provided a milieu for attachment, proliferation, differentiation and migration of cells. Scaffolds with different physical and chemical properties have been developed, including hydrogels based on various components (e.g., hyaluronic acid or fibrin). The advantages of hydrogels are a high liquid content and the ability to retain cells without impairing their functions [11,12]. A fibrin-based gel (or ‘fibrin glue’) has advantages for tissue engineering: (a) both allogeneic and autologous plasma can be used as a source of fibrinogen, (b) gel polymerization is initiated by non-cytotoxic initiators (thrombin, calcium salts) (c) the gel structure is strong enough to ensure reliable retention of cells at the injection site, (d) significant water content in the gel makes possible the introduction of water-soluble biologically active substances into it, (e) fibrin is a biodegradable material [13,14,15]. Fibrinogen-based scaffolds harden by cleaving fibrinogen that can be cryoprecipitated from a patient’s or donor’s blood plasma [16,17,18,19]. The mechanical properties of the fibrin-based gels can be regulated by varying the amount of fibrinogen and fibrinogen:thrombin ratio, as well as by adding additional components, for example, collagen [16,17,20]. However, neither fibrin nor DPSC can be used in standard 3D printing.

The aim of the study was to develop a technology of cell-seeded fibrin gel implant molding that has the same shape and size as the bone defect at the site of implantation. In our study, an anatomical prototype-a mold representing defects of the vestibular and palatal fragments of bone tissue was created by 3D printing. This 3D form was filled with fibrin glue and DPSC suspension. The approach allowed to obtain a cells-containing implant that matches the bone defect, was hard enough to preserve its shape and allowed cell proliferation and migration. In mice with surgically created bone defects, the implants increased the rate of bone remodeling.

## 2. Materials and Methods

All experiments performed in this study received approval from the local ethical committee of North-Western State Medical University (ethical vote No 12, date of issue 12 December 2019).

### 2.1. 3D Printed Anatomical Prototype Molding Form

Spiral computerized tomography (SCT) analysis of alveolar bone structure was carried out with Toshiba Aquilion Prime scanner equipped with Canon Medical Systems’ SEMAR (Single Energy Metal Artifact Reduction), step-1 mm.

Specialized software, 3D Slicer 4.10.2, was used for visualization, segmentation, and 3D reconstruction of teeth hard tissues and alveolar bone. The shape of the bone defect was reconstructed using CT images, and a 3D computer model of the molding form was built. According to this 3D model, an anatomical prototype of vestibular and palatal bone fragments with the defects was made of polylactic acid fibers using fused deposition modeling (FDM) 3D printing technology.

### 2.2. Cell Cultures

Human DPSC were obtained from extracted retained or dystopic 3rd molar of donors (18–27 years old). Pulp was extracted from a pulp chamber after opening the crown, sliced with scissors and/or scalpel and incubated with 2 mL of Collagenases I, IV (0.1% each in saline) on a shaking platform (200 rev/min) at 37 °C for 45 min. Then, 8 mL of saline was added. The tube was centrifuged, and the pellet was transferred into a 25 cm^2^ cell culture flask (TPP, Trasadingen, Switzerland) and cultured in Advanced Stem Cell Medium (HyClone, Logan, UT, USA) supplied with 10% ASCM supplement (HyClone, Logan, UT, USA) and penicillin/streptomycin (Gibco-Thermofisher, Waltham, MA, USA). Cells were cultured under hypoxic conditions (7% О_2_, 5% СО_2_). This protocol is referred to below in the Result section as ‘standard conditions.’ The medium was changed every 3 days. Cells that reached 70–80% confluence were subcultured using standard methods. After they were split (passaged) for the 2nd time, DPSC were taken into the experiments after validation of their viability, immunophenotype (see the Flow cytometry section below), and the absence of *M. hominis*, *M. genitalium*, HIV1, 2, *Treponema pallidum*, HBV and HBC antigens were assessed, karyotyping was provided. The technology proposed in the present publication is aimed at application in humans. Therefore, we tested the biocompatibility of the human fibrin glue and human cells. However, the biological efficacy should be first tested in preclinical studies in animals. In preclinical studies, homological models (i.e., mouse cells for in vivo experiments in mice models if the end product is supposed to be made of human cells intended for application in humans) are recommended by the national regulator [21]. Therefore, for animal studies, mouse DPSC were used. Mice (n = 2) were anesthetized, and 2 molars from 1 side were extracted as described below, and the pulp was processed in the same way as human samples. The only difference was the replacement of Advanced Stem Cell Medium (HyClone, Logan, UT, USA) with DMEM:F12 medium (Biolot, St. Petersburg, Russia) because this medium was more appropriate for mouse cells.

### 2.3. Fibrin Glue

Fibrin glue from autologous platelet-poor plasma (PPP) was prepared as we described earlier [17,18]. Briefly, the cryoprecipitate (with fibrinogen concentration of 14–20 g/L) was used to obtain fibrinogen according to the standard protocols [22,23,24] with some modifications described below [18]. All the procedures were carried out in a “closed system” (i.e., without leaving a sterile system of bags, connecting tubes and syringes). The blood (30 mL) was collected in heparin-lithium Vacuette^®^ tubes, and the PPP was obtained by centrifugation. The PPP sample was collected from the tube using a syringe without opening the tube lid. It was then transferred through a multiple-use needle-less port into a 20 mL section of a double-sectioned cryobag (Macopharma, Duluth, GA, USA). If necessary, the syringe was connected to the port through a 0.22 mkm syringe filter to filter the remained leucocytes and platelets. The bag with PPP was stored at −80 °C. To obtain plasma enriched in fibrinogen (cryoprecipitate), the cryobag was slowly thawed at 4 °C until the frozen PPP became ‘slushy.’ The bag with slushy PPP was pressed, and the thawing fibrinogen-depleted plasma flowed down into the 5 mL ‘satellite’ section of the bag and was removed from it with a syringe. The fraction (cryoprecipitate) that remained in the 20 mL section was enriched in fibrinogen and was used for the fibrin glue. If necessary, the same cryoprecipitate was used for the preparation of glues with standard (60–70 g/L) concentration of fibrinogen using the centrifugation of obtained fibrinogen solution in VivaSpin Turbo tubes with the size of pores 100 kDa (Sartorius, Göttingen, Germany). The lysis of fibrinogen was activated with thrombin (200 U/mL, Baxter, Deerfield, IL, USA) and CaCl_2_ (2 g/L, Mapichem, Baar, Switzerland); ε-aminocaproic acid (2 g/L, Mospharm, Moscow, Russia) was added to decrease fibrinolysis.

DPSC were grown as described above. At the confluency 60–70%, they were detached with 0.25% Trypsin (Thermofisher, Waltham, MA, USA), resuspended in 5 mL of the medium, and centrifuged at 800× *g* for 5 min. Immediately before fibrin glue preparation, they were resuspended to the concentration 2 × 10^6^ cells/mL in 2× thrombin solution stock (400 U/mL thrombin, 4 g/L CaCl_2_; 4 g/L ε-aminocaproic acid in saline).

Cryoprecipitate was thawed and mixed with 2× thrombin solution at 1:1 ratio using the double-syringes-single-needle system Duploject (Baxter, Deerfield, IL, USA) during the filling of the molding form (described above) or wells of a cell culturing plate.

The fibrin clot retraction was evaluated, measuring the size of the clot polymerized in a plate well. Saline was layered above the polymerized gel to avoid its drying. The size of gel was evaluated in 1, 12, 24, 72, 120 and 168 h after polymerization.

### 2.4. Evaluation of Cells Proliferation, Differentiation and Surface Markers Phenotype

DPSC-seeded fibrin gel was transferred into a cell culture medium supplied with ASCM supplement and antibiotics/antimycotics. The gel was incubated at 5% CO_2_ and 7% O_2_ for 7 days, with the medium changed every 3 days. On day 7, all the cells, both growing in gel and those that left the fibrin clot, were harvested, and their quantity, osteogenic differentiation and immunophenotype (set of surface markers) were evaluated.

To remove the cells from the fibrin gel, the clot was cut into small pieces, treated with trypsin and incubated with PPP at 37 °C for 3 h as described by Elnager et al., 2014 [25]. Then, the sample was mixed with cells harvested outside the gel (i.e., migrated from it). It was pipetted and passed through a filter mesh (70 mkm).

The cell quantity and viability were measured with the Luna™ cell counter. The viability was additionally measured by flow cytometry of cells stained with 7-Aminoactinomycin D (7-AAD).

To induce osteogenic differentiation, cells were extracted from the gel and left to grow for 24 h in standard conditions described in the section Cell cultures. In 24 h, the medium was changed to MSCgo™ Rapid Osteogenic Differentiation Medium (Biological Industries, Beit-Haemek, Israel). Cells were grown for 10 days (as recommended by the manufacturer) with medium changed every 3 days. On day 10, cells were fixed and stained with Alizarin Red. The intensity of staining was evaluated both visually and with a spectrophotometer as described in [26].

The immunophenotyping was performed with a Navios flow cytometer (Beckman Coulter, Brea, CA, USA) equipped with semiconductor diode lasers (488 and 638 nm) and a standard set of emission filters (blue laser: 525/40, 575/30, 614/20, 695/30 and 755LP; red laser: 660/20, 725/20 and 755 LP). The following monoclonal antibodies panels (Beckman Coulter, Brea, CA, United States) were used to determine positive and negative markers of mesenchymal stromal cells: CD44-FITC/CD73-PE/CD0-PC5/CD105-PC7 and CD34-FITC/CD117-PE/CD14-PC5/CD45-PC7. An additional panel was used to determine HLA-DR exposition on the cell surface. Gating of fluorescence events was carried out using the viability parameter. The viability was estimated by forward and side scattering along with staining with 7-AAD. In each sample, at least 15,000 “targeted events” (events determined as viable cells) were analyzed.

### 2.5. Bone Defect Animal Model and Cell-Seeded Scaffold Implantation

The animals were anesthetized with a Zoletil–Rometar mix in doses optimized for small animals. In the area of the molars on the right half of the lower jaw, a scalloped incision of the oral mucosa was made on the jaw’s vestibular side with a scalpel # 12 in accordance with the rules of minimally-invasive periodontal surgery [27]. The flap was peeled off with a narrow raspatory, the bone tissue of the alveolar process was perforated with a micromotor spherical bur along the projection of the apex of the molar, then the tooth with the adjacent bone tissue was extracted with luxation movements performed with a clamp.

In control 1 (no implant) group (n = 10), the wound was closed with a catgut suture. In control group 2 (cell-free implants, n = 9), a small piece of cell-free fibrin glue (20 mkl) was implanted into the defect area before closing the wound. In the experimental group (n = 9), the implanted fibrin glue was seeded with mouse DPSC (0.5 × 10^6^/mL).

The animals in all groups were euthanized in 28 days using lethal doses of Zoletil–Rometar mix and subjected to computerized tomography and histomorphometric analysis.

### 2.6. Cone Beam Computed Tomography

CBCT scanning was carried out with a Planmeca^®^ ProMax imaging unit (Finland) using the following parameters: 90 kVp, 4 mA, voxel size 75 μm, FOV 4 mm × 5 mm.

The data were post-processed with a noise reduction algorithm and saved in a standard format (DICOM). The images were further analyzed with built-in functions of Romexis^®^ software. Regions of interest (ROI) were defined in multiplanar reconstruction (MPR) mode with the planes positioned according to the maximal measurements of a bone defect [28].

### 2.7. Histomorphometric Analysis

For histological examination, the implantation sites were excised together with the surrounding tissues. The opposite half of the jaw was excised in the same way and used as a control. All specimens were fixed with 10% formalin buffered solution (рН7.4) for 24 h. Decalcification was performed in a commercial decalcifying solution TBD-2 (Thermo Fisher Scientific, Waltham, MA, USA) for 1–3 days. The readiness of the samples was determined using a sharp needle test. If the needle passed into the bone tissue without crunching, the decalcification was considered complete. Dehydration and embedding in paraffin were performed using an automatic Excelsior AS Tissue Processor (ThermoFischer, Waltham, MA, USA) and commercial medium IsoPREP (Biovitrum, St. Petersburg, Russia). Finally, the specimens were impregnated with HISTOMIX medium (Biovitrum, St. Petersburg, Russia), and the obtained paraffin blocks were sliced into 3 μm thick sections by rotary microtome НМ325 (ThermoFisher, Waltham, MA, USA). The slices were stained with hematoxylin-eosin according to the protocol of the manufacturer (Biovitrum, Russia) and analyzed with transmitted-light bright field AxioLab Zeiss microscope (Carl Zeiss, Oberkochen, Germany).

The morphometric study was carried out using a Carl Zeiss Axio Imager AZ microscope (Germany) supplied with the AxioVision software (version 4.8, Carl Zeiss, Germany) for the analysis of images. To verify the histological changes, histoarchitectonics of adjacent tissues were evaluated.

The morphological characteristics of the studied samples included the following parameters: the degree of fibrosis, the degree of bone remodeling, the severity of the inflammatory reaction and vascularization in the defect zone.

To assess pathomorphological changes in the groups, a modified scale of semi-quantitative assessment was used in points for the following parameters of the severity of the trait: 0—absent; 1—weakly expressed; 2—moderately expressed; 3—prominent.

The analysis was carried out in 10 fields of view in areas oriented in the same plane. The average parameter value for each sample and then the average parameter value for the group were calculated.

### 2.8. Statistical Analysis

Values are means ± standard error. A comparison of mean values between groups was evaluated by two-tailed Student’s t-test using the GraphPad Prizm. *p* values < 0.05 and less were considered significant. The experiments were always performed in triplicates. Any *p*-value < 0.05 is designated with one asterisk.

In the histomorphometric analysis, the data were analyzed using the Kruskal–Wallis test, and *p* < 0.05 was set as the level of statistical significance.

## 3. Results

**Figure 1:**
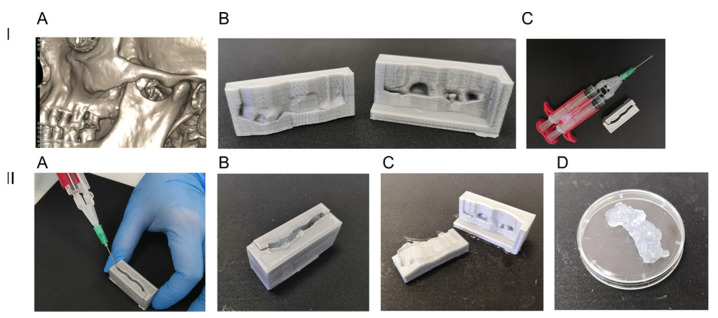
3D molding form and preparation of anatomical cell-seeded scaffold. Panel I. (**A**) a jaw and teeth 3D model reconstructed on CT data; (**B**) vestibular and alveolar parts of a 3D printed molding form; (**C**) an assembled molding form and a Duploject™ application system for fibrin glue application. Panel II. (**A**) Filling the form with fibrin gel. One syringe was filled with cryoprecipitate, the second one with 2× thrombin solution with resuspended DPSC; (**B**) an assembled form filled with DPSC-seeded fibrin gel; (**C**) the form opened after gel polymerization (approx. 1–3 min); (**D**) A DPSC-seeded fibrin gel matching the bone defect.

3D models of jaw and teeth were reconstructed using the segmentation of CT slices (Figure 1A). The models were used for printing molding forms that consisted of two parts corresponding to the alveolar and vestibular sides of the defect (Figure 1B,C).

Printed molding forms consisted of two parts corresponding to the vestibular and alveolar sides of the defect. When the form was assembled, the form could be filled with fibrin gel that corresponded to the area of the defect after polymerization (Figure 1, Panel II).

Thus, the technology can be used for molding a fibrin gel implant matching the bone defect that was scanned by CT.

### 3.1. Human DPSC Grown in Fibrin Scaffolds: Proliferation, Immunophenotype and Osteogenic Differentiation

**Figure 2:**
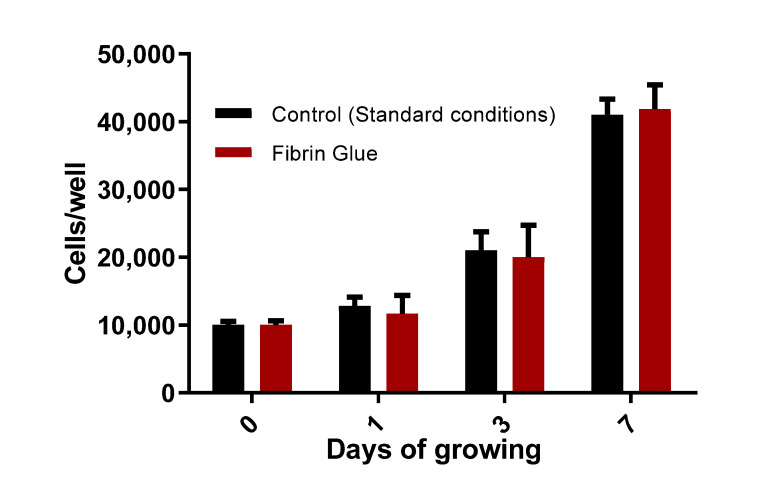
The proliferation of human DPSC grown in fibrin gel and in standard conditions (control). X-axis: days after passaging. Y-axis: the quantity of cells per well. Values are shown as means ± standard error.

The proliferation rate of cells grown in fibrin gel did not differ significantly from the values obtained for cells grown in standard conditions (Figure 2).

DPSC has a set of surface markers (immunophenotype) typical for MSC [5,29]. We evaluated the immunophenotype of the control (grown in standard conditions) and experimental (grown in fibrin glue) cells. Cells of both groups have a similar set of surface markers (Table 1). It is also important that DPSC shared another feature with MSC-DPSC in both of the groups that did not present HLA-DR antigen on their surface. Thus, donor DPSC might be a good candidate for seeding scaffolds when autologous cells are not available due to different reasons.

**Figure 3:**
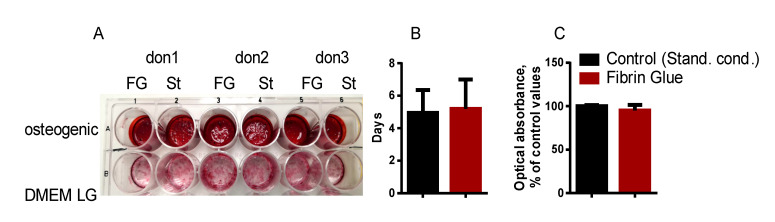
Osteogenic differentiation of DPSC in fibrin gel. (**A**) A plate with differentiated cells (FG-grown in fibrin gel; St-grown in standard conditions) from 3 donors. Cells are fixed and stained with Alizarin Red. The upper row of wells: 14 days of osteogenic differentiation in MSCgo™ Rapid Osteogenic Differentiation Medium; the bottom row of wells: cells grown in DMEM-low glucose medium. (**B**) The rate of first calcificates appearance in cells grown in gel and in standard conditions. Y-axis: the days after the start of osteogenic induction. (**C**) To quantify calcificates, Alizarin Red bound to calcificates was dissolved, and the optical absorbance was measured as described by Bogdanova et al., 2018 [26]. The value obtained for the control cell was set as 100% (Y-axis). The values in the panels (**B**), (**C**) are given as M ± m (means and standard errors).

Osteogenic differentiation is one of the most important features of cells intended for implantation into the bone defect area. Therefore, we studied the osteogenic potential of cells after embedding in fibrin glue. The rate of osteogenic differentiation was similar in control cells and cells after embedding in fibrin gel. The amount of calcificates and the rate of their appearance did not vary significantly between the groups (Figure 3).

**Figure 4:**
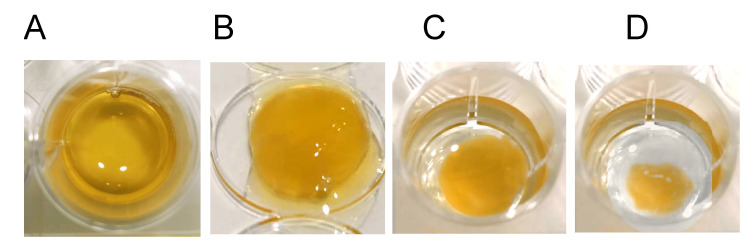
High density (with fibrinogen concentration of >20 mg/mL) fibrin gel clot retraction in vitro. The plasma used for a cryoprecipitate preparation was centrifuged at 12,000× *g* and then filtered to remove platelets, which promote retraction. A fibrinolysis inhibitor, ε-aminocaproic acid, was added to a gel to inhibit the residual components of the plasmin–plasminogen system in the cryoprecipitate. Fibrinolytic components are also important for gel stabilization in vivo. Saline was layered onto polymerized fibrin gel to avoid its drying. Fibrin gel colts are shown in (**A**) 1 h, (**B**) 48 h, (**C**) 120 h, (**D**) 168 h after polymerization.

Clot retraction is one of the feature characteristics of fibrin-based gels. These gels are characterized by a rapid change of their shape due to clot retraction. However, this phenomenon is observed at a low concentration of fibrinogen (less than 10 g/L) [30]. When we used cryoprecipitates with a fibrinogen concentration of 20 g/L obtained from filtered platelet-deprived plasma, as well as a fibrinolysis inhibitor ε-aminocaproic acid, the retraction was not observed during the first 4 days, and after five days, the clot volume decreased by an average of 22.0 ± 3.2%, after 7-by 57.25 ± 4.7% (Figure 4). In low fibrinogen gels (less than 10 mg/mL), 50% retraction was observed within 3 h after polymerization.

**Figure 5:**
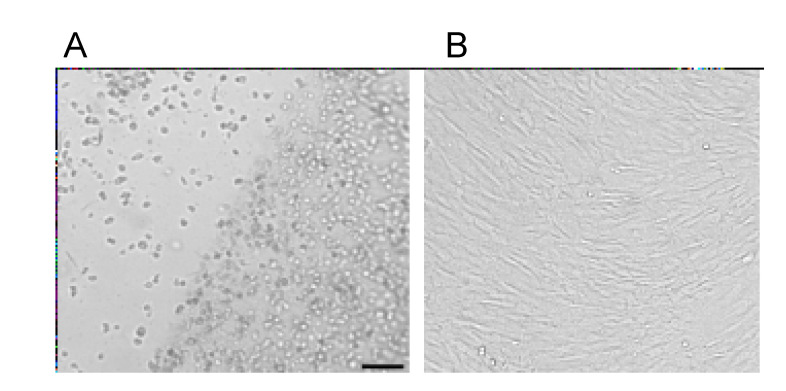
DPSC in fibrin gel (**A**) at day 5 after seeding. Some cells left the gel where cells were grown at a high density. The fibrin gel was removed from the Petri dish, and (**B**) in 3 days, the cells that left the gel expanded to 100% confluency. The scale bar: 100 mkm.

The fibrinogen concentration is an important parameter of cryoprecipitate or any other fibrinogen source used for gel preparation. It determines not only the rheological and mechanical parameters of the clot and its retraction rate but also the size of the pores formed during polymerization between fibrin fibers [31]. The pores allow cells to migrate to/from the scaffold. In our experiments, migration of cells from the fibrin glue was observed (Figure 5), suggesting that the pores of the glue are large enough to allow cells migration from the glue.

### 3.2. Alveolar Bone Defect In Vivo. Influence of Cell-Seeded Implant on Bone Remodeling

Despite the monophyodont dentition and reduced dental formula along with hypsodont incisors, toothless diastema and enamel-free areas on molar surfaces, in general, mouse models can be used in a preclinical study in dentistry [32].

**Figure 6:**
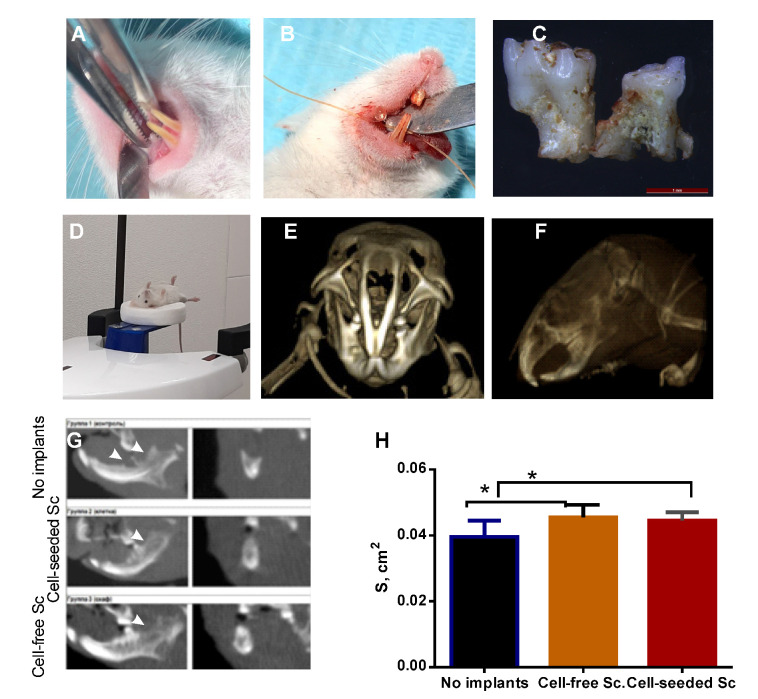
The Cone Beam CT analysis of the bone tissue area at the site of the defect. (**A**) The extraction of 2 molars with surrounding bone tissue to create a defect; (**B**) Wound closing with a catgut; (**C**) Molars extracted a fragment of the alveolar bone (between the roots); (**D**) A mouse in a cone beam CT scanner; (**E**) A Volume Rendering (VR) of a mouse skull, and (**F**) MIP reconstruction; (**G**) MPR images (left: sagittal, right: coronal) of the alveolar bone with a defect (pointed by arrows), in which the different size of the alveolar bone in the no-implant group and the groups with implants is visualized; (**H**) A diagram of the bone tissue area evaluation at the site of the defect in three groups of animals. * *p* < 0.05 vs. no implant group; Y-axis: the area of bone tissue, cm^2^.

The oral cavity of the mice was examined under anesthesia. One or two (in case of fusion) molars were removed from the right side of the lower jaw, and a bone defect was created at the same time (Figure 6A–C,G). During the morphological examination, an almost complete absence of the pulp in the pulp chamber and root canals was revealed; however, in the area of the apical part of the root, a sufficient quantity of dental pulp was extracted and used for expansion in vitro. Periodontal cells were also present in extracted teeth surrounding tissues and used for seeding the fibrin gel before implantation. Some of the extracted molars are shown in Figure 6C, and bone tissue is visible between the roots. Thus, a bone defect was created, which was subsequently filled with a fibrin glue seeded with allogeneic murine DPSC from 2 mice that had been operated 2 weeks prior to the animals of control and experimental groups. In experiments on mice, the molding technology was not used due to the small area of the defect. A drop of fibrin glue was put into the defect area using the Duplojet™ application system. The surgical wound was then closed with a catgut suture, and in 28 days, the animals were put to sleep and subjected to CT, followed by the extraction of the lower jaw for histological examination.

CT is often used to evaluate the density of bone tissue in the area of the defect. Taking into account the ambiguity of the literature data on the correspondence of Cone Beam CT gray values (CBCT GV) to Hounsfield units (HU) [33], low signal-to-noise ratio (SNR) associated with high spatial resolution and small ROI, we decided that measuring the density characteristics in mouse bone defect is not reliable. For an objective evaluation of bone tissue defects, the area of the bone defect sections was measured (Figure 6G,H). Tooth extraction usually involves alveolar bone loss and reduction in height and width of the remaining alveolar socket, owing to the physiological bone resorption [34]. Therefore, a value of the total bone volume is an important parameter characterizing bone repair. The area of bone tissue was significantly higher in groups where the fibrin gel was implanted. However, no significant difference was revealed by CBCT between the groups with cell-seeded and cell-free fibrin implants (Figure 6H), probably due to the modality limitations associated with a small size of the evaluated area in experiments on such small animals as mice.

**Figure 7:**
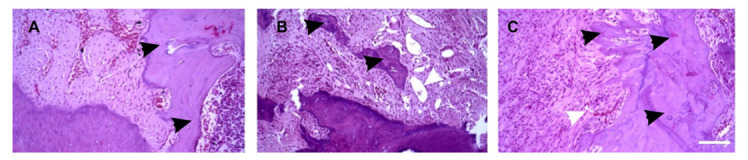
Bone remodeling in hematoxylin-eosin-stained sections of bone defect: (**A**) with no gel implanted, (**B**) cell-free implant and (**C**) cell-seeded implant (white arrows-vessels, black arrows-bone remodeling tissue). Scale bar: 100 mkm.

CBCT revealed the difference between the groups with and without fibrin implants but not between the groups with and without DPSC added to a gel. A histological examination was performed to analyze the process of bone remodeling in the area of the defect (Table 2 and Figure 7). During the examination at the macroscopic level, the search for the bone defect was carried out, focusing on the position of the bone callus of different sizes and degrees of maturation. In all the animals, the bone defect was repaired by forming both areas of fibrous tissue and areas of bone callus with randomly newly formed bone beams.

Prominent changes in histoarchitectonics in the defect area and surrounding tissues were observed in the group of animals with implanted cell-seeded fibrin gel. In this group, bone remodeling and fibrosis of varying degrees of the surrounding soft tissues were observed in a larger area as compared to the group with cell-free scaffolds implanted (Table 1). The absence of capsules along with massive macrophage infiltration proved the integrity of the scaffold materials. Vascularization is an important parameter contributing to the acceleration of the regeneration process. Vascular growth leads to improved oxygenation of damaged tissues and promotes both accelerated regeneration and migration of white blood cells to the site of inflammation for faster and more successful lysis of tissue and cellular detritus. In this group, a larger number of vessels at the edges of the defect was observed as compare both to no-implant and cell-free groups. The samples of the animals with implanted cell-seeded gel had a greater number of bone islets, which occupied a larger area (Figure 7). In the cell-free fibrin group, no significant differences in the number of bone islands were revealed as compared to the control group. However, in this group, the number of vessels in the defect area was higher compared to the animals with an untreated bone defect.

Some samples showed moderate polymorphocellular inflammatory infiltration, which is probably more associated with the presence of bacterial infection in the oral cavity of animals. In 2 cases in the cell-seeded gel group and 1 case in the cell-free group, local areas of bone tissue with a perifocal epithelioid-giant cell reaction were observed. This reaction was also observed near the remains of the suture material.

Thus, fibrin glue itself promotes vascularization and has some influence on the activation of the regeneration process in the area of the bone defect. Nevertheless, the presence of DPSC increased the rate of bone remodeling and the appearance of bone tissue. The combination of DPSC and fibrin gel can be used in implants to promote bone regeneration. The 3D printing technology makes it possible to mold the implants matching the area of the defect. Implants shaped to match the defect are more effective in regenerative periodontology and dentistry because of more tight adhesion to the defect edges.

## 4. Discussion

We have shown the technical feasibility of creating a DPSC-seeded fibrin-based scaffold, with the shape exactly corresponding to the anatomical defect of the jawbone. 3D printing technologies are becoming an integral part of tissue engineering. The main advantage of the approach is the fast and cheap synthesis of a personalized anatomical prototype matching the shape of a defect or a replaced tissue fragment [35]. Methods based on 3D printing are currently being developed in dentistry and maxillofacial surgery [36,37]. However, the most common approach described in published works is the printing of the scaffold itself but not the molding form for it [38]. In this case, cells or cell sheets are layered onto the scaffold surface. Otherwise, the range of materials and printing technologies is limited to the materials and technologies of printing that do not damage the cells in implants [37]. Using molding forms allows using a wider range of materials, for example, fibrin glue, which is not suitable for 3D printing. In addition, the method of molding forms facilitates the introduction of biologically active substances and cells into the scaffold. The combination of fibrin-based scaffolds and DPSC can improve the efficacy of both of them. Fibrin glue protects cells from oxidative stress, keeps a significant portion of cells at the site of implantation without changing their viability and without interfering with the secretion of paracrine factors [31,39].

The properties of the scaffold at a fibrinogen concentration of 20 mg/mL allow cells to migrate, at least from it (Figure 5). Earlier, we showed the possibility of cell migration from the adhesive at low concentrations of fibrinogen in the adhesive [17]. Salem et al. showed that the migration of cells from the gel was observed at a fibrinogen concentration of up to 20 mg/mL [31]. The main factor preventing cell migration is a change in the pore diameter but not in the thickness of fibrin fibers [31]. In our work, we used fibrin glue containing a fibrinolysis inhibitor-ε-aminocaproic acid. This fibrin glue has an increased pore size [40].

Fibrin-based scaffolds are non-toxic for both a patient and the transplanted cells: they are made from components approved for clinical use (thrombin, aminocaproic acid, calcium chloride) and autologous patient plasma. Being ectomesenchymal cells with mesenchymal phenotype, DPSC does not carry HLA class II antigens on their surface (Table 1) and has a very low level of HLA class I expression. The low quantity of HLA antigen on the DPSC surface results in a very low level of immunogenicity. The cells do not cause the proliferation of alloreactive T-lymphocytes both before and after differentiation [41,42,43]. Given the composition of the cell-seeded fibrin gel and the low immunogenicity of DPSC, we suggest that the DPSC-seeded scaffold is biocompatible. In our experiments on the mouse model, we did not observe an immune reaction after implantation of a gel with mouse allogeneic DPSC and human cryoprecipitated plasma.

In our studies, human DPSC retained their viability, immunophenotype and the ability for osteogenic differentiation after embedding in a fibrin-based gel. After the implantation, we observe the increase of the bone tissue volume in the defect area. Periodontal stem cells and DPSC have the ability to restore periodontal and bone defects [44,45]. Fibrin glue itself accelerates bone tissue repair since it mimics the formation of a blood clot in the damaged area, which is a natural stimulator of reparative processes in bone tissue [46,47]. It has been shown that the use of mesenchymal stromal cells in combination with fibrin gel accelerates the restoration of the alveolar bone [48]. Thus, the 3D-printing method for creating a casting mold that is filled with DPSC and components of the gel is applicable for reparative osteogenesis and the elimination of the alveolar bone tissue defects in the methods of tissue regeneration, the outcome of which should be the restored volume of bone tissue necessary for successful dental prosthesis implantation.

The resorption of bone tissue occurs at the area of the defect after tooth extraction [49]. The process of resorption involves the activation of osteoclasts and a change in the activity of osteoblasts due to the increased production of pro-inflammatory cytokines and mediators by periodontal tissues-interleukin-1, tumor necrosis factor α, interferon γ, bone morphogenetic proteins, prostaglandin-E2, matrix metalloprotease and others [50]. These cytokines attract macrophages, monocytes and lymphocytes. DPSC in fibrin glue did not lose the ability for osteogenic differentiation (Figure 3). Various researchers have proven that stem cells of the oral cavity, including DPSC, are involved in the repair of damaged bone tissue. A high osteogenic potential of DPSC has been described [5,9]; therefore, their use for bone tissue repair is a promising method of translational medicine.

The terms of fibrin matrices biodegradation vary depending on the gel composition from 3 to 21 days [40,51,52]. We observe the residual gel clot at the site of the defect in histological samples obtained 28 days after implantation. However, depending on the nosological form of the disease, the timing of biodegradation should be extended. In some cases, it is necessary to extend the time of scaffold biodegradation up to 2 months. To prolong the time of scaffold degeneration, it seems promising to add another well-studied material to fibrin glue, such as collagen [53].

## 5. Conclusions

The technology of 3D-printed forms for molding anatomical implants can be used to prepare implants using components that are not suitable for 3D printing. Fibrin glue is a sufficient material for scaffolds with good mechanical characteristics. The cells embedded in the fibrin glue maintain their viability, immunophenotype and osteogenic potential. Both fibrin glue and DPSC improve bone repair. This technology can be used for bone tissue repair in dentistry and maxilla-facial surgery.

## Figures and Tables

**Table 1:** Immunophenotype of DPSC embedded in fibrin glue and grown in standard conditions.

Surface Antigen	DPSC Grown in Fibrin Gel *	DPSC Grown in Standard Conditions *
CD90	99.7 ± 0.52	99.3 ± 0.8
CD105	99.1 ± 1.1	99.8 ± 0.85
CD44	99.7 ± 0.82	98.9 ± 1.2
CD73	98.2 ± 0.84	98.8 ± 0.5
CD45	0.1 ± 0.07	0
CD34	0	0
CD14	1.2 ± 0.0.4	1.0 ± 0.2
CD117	0.4 ± 0.24	0
HLA-DR	0	0

* Values are given as means and standard errors.

**Table 2:** Histomorphometric characteristics of samples.

Measured Parameter	Group			*p*
	No Implants	Cell-Free Fibrin Glue	Cell-Seeded Fibrin Glue	
Inflammation, Me [Q_1_–Q_3_]	0 [0, 2]	1 [0, 2]	2 [2, 2]	0.203
Fibrosis, Me [Q_1_–Q_3_]	2 [2, 3]	2 [2, 2]	3 [2, 3]	0.243
Remodeling of bone, Me [Q_1_–Q_3_]	1 [1, 3]	1 [1, 2]	2 [2, 2]	0.193
Vessels Me [Q_1_–Q_3_]	1	3	2	0.198
